# Notes on Medical Inspection of School Children

**Published:** 1908-08-22

**Authors:** 


### Public Health and Hygiene.

### NOTES ON MEDICAL INSPECTION OF SCHOOL CHILDREN.

The thoroughness with which the medical inspection of school children is being carried out in this country varies greatly as yet in different districts. In some, the authorities have only arranged for the minimum inspection to be carried out by local practitioners on so many afternoons a week; in others, full time inspectors have been appointed, and the cases are followed up to their homes by a special school nurse. The saving in attendance grants has gone a long way towards paying for the services of the nurse, while the amount of good done by her, directly and indirectly, will certainly be of the greatest benefit to the community. As regards the actual inspection, the lines suggested by the Board of Education have, in the main, been universally adopted, and the 5 in. by 8 in. card has been found the most convenient. It would have been wise to have allowed for more frequent weighing and measuring of the children; a yearly record would form a useful indication of progress, and this part of the inspection is always the most appreciated by the children, the parents, and the teachers. No doubt in the future the metric system will be adopted to the great benefit of all concerned.

The attendance of parents is said to be disappointing, though their attitude towards the inspection is favourable. In the urban district in which the writer works, only about 10 to 15 per cent. of the mothers of the older children attend for the interview, and about 25 per cent. of the infants are accompanied by their mothers. As the main reason for desiring their attendance is for the purpose of eliciting the nature of the child's previous illnesses, it has been found necessary to send out with the official notice of the date of inspection, a paper embodying the six lines on the front of the card and requesting affirmative or negative replies to the several items. These papers are returnable by the child on the following day. They are sent out to the parents of all the younger children, but those leaving are instructed to make verbal inquiries on these points if possible.

Lack of a proper room is an oft-recurring difficulty, and often a side class-room has to be turned out; some derangement is thus inevitable. Occasionally it has been found advisable to conduct sight-testing in the open air in order to secure the requisite distance of 6 metres. It is well to remember that the children soon get to know by heart the letters of the last line, and it is therefore necessary to have in the room only one child at a time, and to keep those done separate from those about to be examined. Two or three different sets of type-cards are necessary.

In examining the fauces properly, some form of tongue depressor will be found indispensable; and it is, of course, highly desirable that each child should have a separate one. Some inspectors use thin slips of wood, such as gardeners' labels, which can be thrown away, but a most useful instrument can be made out of a slip of galvanised iron or of stiff zinc-sheeting, 7 in. by $\frac{3}{4}$ in., bent in the middle to a right angle. These are cheap, easily washed or boiled, and most efficient. They allow a clear view, as the hand is out of the way. About thirty will be found ample to allow of one for each child in a day's inspection, and many children can be examined without using a tongue depressor at all.

It has not been thought necessary to have cards differently coloured for those children who have been found defective, either through some temporary or permanent fault; the boxes, in which the cards are indexed and filed, allow of a grouping of such cases in the "special" section. A child cannot usually be put down as defective until after its examination, and then a card will have been already filled up for it, so that a special card would entail much extra writing. It will be found useful to have one corner of the card snipped off; this will facilitate shuffling and ensure that the cards are in proper position for speedy reference.

